# Genetic Polymorphisms: A Novel Perspective on Acute Pancreatitis

**DOI:** 10.1155/2017/5135172

**Published:** 2017-12-03

**Authors:** Yong Chen, Chao Lian Xie, Ran Hu, Cheng Yi Shen, Mei Zeng, Chang Qiang Wu, Tian Wu Chen, Chen Chen, Meng Yue Tang, Hua Dan Xue, Zheng Yu Jin, Xiao Ming Zhang

**Affiliations:** ^1^Sichuan Key Laboratory of Medical Imaging and Department of Radiology, Affiliated Hospital of North Sichuan Medical College, Nanchong, China; ^2^Sichuan Key Laboratory of Medical Imaging and Department of Pathophysiology, Affiliated Hospital of North Sichuan Medical College, Nanchong, China; ^3^Biology Group, North Sichuan Medical College, Nanchong, China; ^4^Sichuan Key Laboratory of Medical Imaging, North Sichuan Medical College, Nanchong, China; ^5^Radiology Department, Peking Union Medical College Hospital, Chinese Academy of Medical Sciences, Beijing, China

## Abstract

Acute pancreatitis (AP) is a complex disease that results in significant morbidity and mortality. For many decades, it has compelled researchers to explore the exact pathogenesis and the understanding of the pathogenesis of AP has progressed dramatically. Currently, premature trypsinogen activation and NF-*κ*B activation for inflammation are two remarkable hypotheses for the mechanism of AP. Meanwhile, understanding of the influence of genetic polymorphisms has resulted in tremendous development in the understanding of the advancement of complex diseases. Now, genetic polymorphisms of AP have been noted gradually and many researchers devote themselves to this emerging area. In this review, we comprehensively describe genetic polymorphisms combined with the latest hypothesis of pathogenesis associated with AP.

## 1. Introduction

Acute pancreatitis (AP) is an inflammatory disease of the pancreas with an annual incidence ranging from 13 to 45/100,000 persons [1]. Approximately 80% of the cases of AP are mild and self-limited, while up to 20% can be complicated [2]. The most frequent causes of pancreatitis in adults are common bile duct stones and alcohol abuse [3]. Approximately 15–25% of cases of pancreatitis are idiopathic. Despite decades of research, the exact pathogenesis of AP remains to be elucidated.

In the past few years, significant progress in elucidating the mechanisms and the genes involved in AP has been made. Currently, AP is considered a multifactorial disease with contributions from environmental, metabolic, and genetic factors. Rather than considering them in isolation, genetic variants should be considered as cofactors interacting with other causes in AP because of the reversible course of the disease in most cases [4]. Accurate prediction of the development of AP by genetic analysis can contribute to timely intervention and targeted therapy.

**Figure 1:**
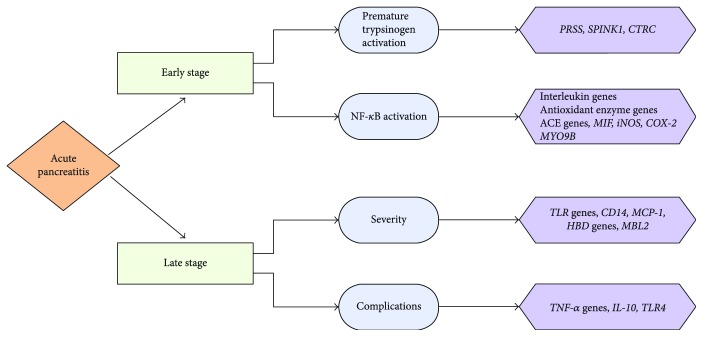
The relationship between different pathophysiological processes implicated in AP and susceptibility genes is concluded as follows: PRSS1: cationic trypsinogen; SPINK1: serine protease inhibitor Kazal type 1; CTRC: chymotrypsin C; ACE: angiotensin-converting enzyme; MIF: migration inhibitory factor; iNOS: inducible nitric oxide synthase; COX-2: cyclooxygenases 2; MYO9B: myosin IXB; TLRs: toll-like receptors; MCP-1: monocyte chemoattractant protein-1; HBDs: human *β*-defensin 2; MBL2: mannose-binding lectin 2; TNF-*α*: tumor necrosis factor-*α*; IL-10: interleukin 10; TLR4: toll-like receptor 4.

In this review, we divided the susceptibility genes into several categories to describe the impact of genetic polymorphisms, and we combined this information with the latest hypotheses for the pathogenesis associated with AP for a better understanding of the disease (see in Figure 1).

## 2. The Current Understanding of the Pathophysiology of AP

Physiologically, trypsin is activated by trypsinogen and then transported to the duodenum, resulting in the activation of the digestive enzyme cascade. For decades, it was believed that premature trypsinogen activation was the central pathogenic event of pancreatitis. This hypothesis is known as the trypsin-centered theory [5, 6]. However, the emerging evidence that both premature trypsinogen activation and inflammatory signaling, two important early events, are sufficient to induce acinar cell damage and result in AP has challenged this theory. In addition, premature trypsinogen activation alone is not sufficient to explain the occurrence of systematic complications during AP, suggesting that the inflammatory signaling pathway may play a critical role in exacerbating the disease [7]. The activation of NF-*κ*B, which acts as a key inflammatory pathway in the pathogenesis of AP, is likely an independent event paralleling premature trypsinogen activation [7, 8].

## 3. Early-Stage AP: Two Early Events in Acinar Cells

### 3.1. Premature Trypsinogen Activation

Physiologically, plethoric trypsin is stored as inactive zymogen precursors to avoid autodigestion in the acinar cells. Cationic trypsinogen and anionic trypsinogen (encoded by *PRSS1* and *PRSS2*, resp.) are the most abundant forms of trypsinogen in the pancreas [9]. When zymogens are transported to duct cells, the bicarbonate-rich fluid secreted by duct cells flushes them out of the pancreas into the duodenum. This process depends on cystic fibrosis transmembrane conductance regulator (CFTR), which can transport chloride and bicarbonate. The opening of CFTR channels hinges on the activation of sensors expressed on duct cells. The whole process depends on calcium signaling, which can stabilize the calcium-binding site of the trypsinogen activation peptide (TAP) to activate trypsin in acinar cells and facilitate the secretion of fluid and bicarbonate in duct cells [10, 11]. A sustained global rise of calcium from the endoplasmic reticulum (ER) membrane and the plasma membrane denotes a detrimental response in AP [10]. An imbalance in cathepsin B and cathepsin L, which activate trypsinogen and degrade trypsin, respectively, can result in colocalization of lysosomes and zymogens and retard the progress of autophagy [12]. In addition, low pH, oxidative stress and redox signaling, mitochondrial dysfunction, and ER stress have been implicated in the premature activation of trypsinogen in AP [7, 8, 13]. Physiologically, autolysis by trypsin and chymotrypsin C (CTRC) and inhibition of trypsin by the trypsin inhibitor (serine protease inhibitor Kazal type 1 (SPINK1)) can protect against trypsin activation [14–16]. Genetic variants of these pancreas-specific genes may change the function and expression of the encoded proteins in AP. Focusing attention on these and other pancreas-specific genes may benefit our complete understanding of premature trypsinogen activation and provide targets for future therapy based upon this mechanism.

### 3.2. Genes Associated with a Susceptibility to Premature Trypsinogen Activation

#### 3.2.1. PRSS1-PRSS2 Locus

Cationic trypsinogen (PRSS1) is the main component of trypsinogen and more easily activated. *PRSS1* is located on chromosome 7q34, and a gain-of-function mutation has been found to increase the propensity for premature trypsinogen activation [17]. A T to C substitution at rs10273639, in the noncoding region of *PRSS1-PRSS2* locus (near the *PRSS1* gene), is associated with recurrent pancreatitis and CP by influencing the expression of *PRSS1* [4, 18]. A Russian study revealed that the C allele at the locus is strongly associated with AP accompanied by excessive alcohol consumption and smoking in male patients [19]. There are several issues deserving attention. First, the epidemiology shows that the frequency of men with alcohol-related pancreatitis is higher than that of women [1]. Further investigation of the effects of sex difference and alcohol consumption on genetic variants is needed. Second, the interaction between genetic polymorphisms and environmental factors needs to be elucidated. Excessive and long-term ethanol intake may be detrimental to the defensive mechanisms (e.g., the antioxidant defense system) against acinar cell injury. Smoking is strongly interactive with alcohol abuse as a cofactor that triggers chronic and recurrent pancreatitis. The mechanism of the rs10273639 effect on ethanol and smoking for the onset of AP needs elucidation especially strong linkage disequilibrium found between rs10273639 and other polymorphisms [20, 21].

#### 3.2.2. SPINK1

SPINK1 acts as a protective protein against trypsinogen activation by inhibiting trypsin with a specific target substrate. *SPINK1* N34S (rs17107315), an A>G transition in exon 3, has previously been shown to correlate with chronic, idiopathic, and recurrent pancreatitis [22–24]. Although the mechanism of the *SPINK1* N34S to pancreatitis is unclear, it was thought that the mutation leads to a diminished capacity to inhibit trypsin [23]. The first research which reported that this polymorphism is associated with AP was performed in Finland [25]. By analyzing 371 patients and 459 controls, the investigators revealed that *SPINK1* N34S increased the susceptibility to AP (*P* < 0.0001). Two later studies from Poland and India confirmed the result (*P* = 0.006 and 0.03, resp.) [26, 27]. However, this polymorphism is common in the general population at a frequency of approximately 2% [28], which suggests that it plays the role of a modifier rather than a causative factor. However, the function of *SPINK1* N34S needs further investigation especially previous study revealing that it is associated with recurrent AP rather than sentinel AP [23]. Meanwhile, emerging evidence shows overexpression of SPINK1 is involved in several cancers acting as an acute-phase reactant, a growth factor, and a regulator of apoptosis [29], which may provide a novel perspective in AP not merely a trypsin inhibitor.

#### 3.2.3. CTRC

CTRC is involved in the autoactivation of trypsinogen by two seemingly paradoxical pathways. On the one hand, by processing the TAP, CTRC stimulates the autoactivation of trypsinogen. On the other hand, CTRC regulates the hydrolysis of trypsinogen by the cleavage of the calcium-binding site of trypsinogen. A polymorphic, synonymous variant of *CTRC*, p.G60=, has been reported to increase the risk of CP [30, 31]. In a recent paper, it was reported that this mutation may mediate the occurrence of AP (*P* = 0.015) [32]. The mechanism by which the synonymous variant influences the occurrence of AP may involve posttranscriptional processing. A more interesting phenomenon is the predisposition for a severe disease in a limited number of patients with this polymorphism and *SPINK1* N34S, which may indicate the important role that genetic variants play in AP and that multigene factors may interact with each other to exacerbate the disease.

### 3.3. The Inflammatory Signaling Pathway: NF-*κ*B Activation

Pathologic calcium overload and the activation of protein kinase C (PKC) isoforms are involved in NF-*κ*B activation. Initially, signaling of proinflammatory mediators in vivo stimulates I*κ*B kinase (IKK) to develop the active NF-*κ*B dimer p50/p65 by phosphorylation and ubiquitination. Subsequently, active NF-*κ*B travels to the nucleus to bind DNA response elements, resulting in the upregulation of proinflammatory cytokine genes [33]. Proinflammatory mediators, mainly tumor necrosis factor *α* (TNF-*α*) and interleukin-1 (IL-1), which are considered to be first-line cytokines, activate the NF-*κ*B signaling pathway, forming a positive feedback loop and subsequently elevating the level of other cytokines and chemokines to cause acinar cell damage under stress.

### 3.4. Susceptibility Genes in the Inflammatory Signaling Pathway

#### 3.4.1. Interleukins

The interleukin family plays a vital role in the progression of AP. IL-1 and TNF-*α*, as described above, are the first proinflammatory mediators released, and they accelerate a series of inflammatory responses during AP. IL-6 and IL-8, both proinflammatory cytokines, are induced by the two first-line cytokines to exacerbate the disease. Separately, IL-10 acts as an anti-inflammatory mediator and is beneficial in the early stage of AP by suppressing the proinflammatory cytokines but is perhaps detrimental in the late stage of AP, especially when bacteria invade from the intestine. In 2000, one study found that three of five polymorphisms in intron 2 (alleles 1, 2, and 3 from a variable number of tandem repeat units) of the *IL-1RN* gene, which encodes the IL-1-receptor antagonist, seemed to determine the severity of AP and the susceptibility to idiopathic acute pancreatitis (IAP) [34]. However, one year later, a British paper did not find any evidence that *IL-1RN* was associated with SAP [35], but the researchers found that the IL-1*β*/IL-1RN ratio was low, implying an elevation of IL-1RN. Although the effect of the polymorphism is controversial, the two studies together showed that IL-1RN is an anti-inflammatory mediator that may be detrimental in severe cases because of its excessive elevation. In addition, the studies showed that IL-1*β* polymorphisms are not associated with AP [34–36]. A meta-analysis that mainly focused on interleukin gene polymorphisms showed that the *IL-8*-251T/A (rs4073) polymorphism increased the risk of AP [36]. In addition, another study indicated that the *IL-10*-1082A/G gene polymorphism led to the onset of AP in Chinese patients (*P* = 0.007) [37]. The interleukin family includes the most abundant cytokines implicated in AP; however, the inconsistent results of these studies make it unclear whether these genes play a critical role on AP by regulating related proteins or they are just bystanders in the progress of AP.

#### 3.4.2. Antioxidant Enzyme Genes

Oxidative stress is considered a crucial mediator during AP, and it appears to exert a paradoxical role. Injured acinar cells promote the generation of reactive oxygen species (ROS), resulting in less severe disease progression by triggering apoptosis. Interestingly, inhibiting ROS in experimental models propagates the local and systemic inflammation by releasing the amount of inflammatory substances needed to induce necrosis of acinar cells. Genetic variants of the glutathione S-transferase (*GST*) family and caspase, both of which are antioxidant enzymes, are reported to be involved in AP. The GST family, which catalyzes the binding of glutathione (GSH) to free radicals, mainly includes 4 subclasses (namely, A, M, P, and T) in the pancreas. GSH is a scavenger of ROS, and the depletion of glutathione is accompanied by AP. Several polymorphisms of the *GST* genes associated with the occurrence or severity of AP have been reported by different investigators [38, 39]. Caspase is a key initiator of apoptosis, which may protect acinar cells against necrosis. The *CASP9* Ala28Val polymorphism (rs1052571), located in an intron of caspase-9, decreases the probability of a mild form of AP [39]. The complex pathophysiology of oxidative stress, including pathogenic apoptosis in AP, requires elucidation before a targeted treatment can be developed.

#### 3.4.3. The Gene Coding for Angiotensin-Converting Enzyme

The renin-angiotensin system (RAS) is responsible for maintaining blood pressure and electrolyte balance. The angiotensin-converting enzyme (ACE) is implicated in RAS for converting angiotensin I to angiotensin II, which is an effective vasoconstrictor. Pancreas-specific RAS is reported to be involved in AP because it causes chronic hypoxia in experimental animals [40]. RAS can also activate immune cells to release proinflammatory cytokines, leading to the activation of the inflammatory signaling pathway. A meta-analysis combining several studies revealed an insertion/deletion (I/D) polymorphism of the *ACE* gene that increases the risk for AP [41]. Furthermore, a study that combined 544 patients from 3 countries showed that this polymorphism is associated with alcohol-related AP (*P* = 0.03) [42]. Persistent ethanol intake can impair mitochondria and gradually result in chronic hypoxia, which may explain the mechanism of the effect of this polymorphism on AP.

#### 3.4.4. Macrophage Migration Inhibitory Factor (MIF)

In addition to TNF-*α* and IL-1, MIF plays a proinflammatory role in AP, especially in severe cases [43]. Previous studies have shown that an elevated serum level of MIF can be an important marker for predicting the severity of pancreatic necrosis in AP [43, 44]. However, by analyzing 164 AP patients in the UK, Makhija et al. [45] showed that the *MIF*-173 (rs755622) polymorphism correlated with AP rather than with SAP and that the *MIF*-173C allele was significantly increased in AP patients (patients 58/320, 18.1% versus controls 47/394, 11.9%; *P* = 0.025). More studies are needed to elucidate the mechanism underlying the effects of *MIF* gene polymorphisms on AP.

### 3.5. Inducible Nitric Oxide Synthase (iNOS)

Nitric oxide (NO) is an important signaling molecule that exhibits dual and opposite influences on the generation of ROS. The negative regulatory role of NO acting as a cytotoxic agent in pathological processes can result in inflammatory disorders, including AP [46]. One of the three nitric oxide synthase (NOS) isoforms, iNOS, catalyzes the conversion of L-arginine to NO [46]. It has been reported that the elevation of iNOS expression in the lung is associated with acute lung injury (ALI) secondary to acute necrotizing pancreatitis (ANP) [47]. Based on this report, investigators from Turkey studied three *iNOS* single-nucleotide polymorphisms (SNPs) in AP patients and found that *iNOS* Ser608Leu (rs2297518) was correlated with a risk of AP (*P* = 0.002) [48]. In contrast, a study of a Romanian population did not find any association between *iNOS*-2087A>G (rs2297518) and the risk of developing AP [49].

### 3.6. Cyclooxygenase-2 (COX-2)

Cyclooxygenase (COX), also known as prostaglandin-endoperoxide synthase (PTGS), is at the core of prostaglandin synthesis regulation. Prostaglandin can induce inflammation, promote vasodilation, and increase capillary permeability. Previous studies have confirmed that COX-2, an isoform of COX, is associated with the development of AP, and inhibiting COX-2 by specific nonsteroidal anti-inflammatory drugs (NSAIDS), for example, indomethacin and diclofenac, may ameliorate the severity of AP [50]. Based on the vital role of COX-2, investigators from Turkey studied 7 *COX-2* SNPs in the local population, and they found that the rs5275 polymorphism in the 3′-untranslated region of the *COX-2* gene was correlated with susceptibility to AP (*P* = 0.02) [51].

### 3.7. Myosin IXB (MYO9B)

*MYO9B* encodes an unconventional myosin molecule that participates in maintaining the gastrointestinal mucosal barrier function possibly by affecting the assembly and positioning of tight junctions [52]. It has been reported that polymorphisms in this gene are relevant to susceptibility to celiac disease and ulcerative colitis [52, 53]. By analyzing a combined cohort of Dutch and German patients with AP, Nijmeijer et al. [54] revealed that two polymorphisms in *MYO9B* were related to susceptibility to AP after correcting for multiple testing. The nonsynonymous variant rs1545620 exhibited a tighter relationship with AP, especially in the Dutch cohort (rs7259292, *P* = 0.0031; rs1545620, *P* = 0.0006).

## 4. Late-Stage AP

As described above, two events can trigger AP, but NF-*κ*B activation may contribute to the progression to a severe form of the disease. The inflammatory cascade activated by NF-*κ*B may result in a poor prognosis or even death. Initially, cell necrosis releases damage-associated molecular patterns (DAMPs), which activate the inflammasome to enhance the disease. Furthermore, the DAMPs are recognized by pattern recognition receptors (PRRs), which mistake DAMPs for pathogen-associated molecular patterns (PAMPs) present on microbes. The whole process is involved in the innate immune system in a condition called sterile inflammation. DAMPs and proinflammatory mediators, including TNF-*α* and IL-1, result in leukocytes infiltrating the site of inflammation. In this process, invasive leukocytes secrete various inflammatory mediators that accelerate the exacerbation of the disease [55, 56]. For example, acinar cells secrete chemokines, including IL-8, which recruit neutrophils to the site of inflammation. Monocytes secrete proinflammatory cytokines, including IL-1 and TNF-*α*. In addition to cytokines, immune cells secrete other inflammatory mediators, including prostaglandin, thromboxane, platelet activator factor (PAF), free radicals, and nitric oxide. These substrates increase vascular permeability, microvascular disturbances, and coagulation abnormalities, which may result in gut barrier dysfunction with increased intestinal permeability [33]. Increased intestinal permeability contributes to death in SAP patients due to sepsis caused by bacterial translocation [57], a response termed the second infection or infectious inflammation. An overwhelming release of cytokines and inflammatory mediators leads to multiorgan dysfunction syndrome (MODS) and activation of compensatory anti-inflammatory response syndrome (CARS), the latter leading to immunosuppression by releasing anti-inflammatory cytokines, which further increase susceptibility to bacterial infection [33].

### 4.1. Susceptibility Genes Contributing to the Severity of AP

#### 4.1.1. Toll-Like Receptors (TLRs)

TLRs are a type of PRR that mediate the innate immune response, resulting in a series of inflammatory events. There are cell surface TLRs and intracellular TLRs, with the former mainly recognizing microbial membrane components and the latter recognizing nucleic acids derived from bacteria, viruses, and the host [58]. By activating TLR signaling pathways, TLRs orchestrate innate immunity and adaptive immunity. Given the importance of TLRs, their impact on AP has been investigated. Japanese investigators discovered a microsatellite polymorphism in intron 2 of the *TLR2* gene, specifically, shorter guanine-thymine (GT)_*n*_ repeats in the locus, with a strong correlation with susceptibility to and severity of AP ((GT)_*n*_* n* ≤ 16, *P* < 0.001 and (GT)_*n*_ 17 ≤ *n* ≤ 22, *P* < 0.001) [59]. Matas-Cobos et al. [60] analyzed 11 polymorphisms of *TLR* genes and suggested that *TLR3* rs3775291 and *TLR6* rs5743795 might affect AP in opposite ways; that is, the CC genotype of rs3775291 might increase the risk of severe pancreatitis, and the GG genotype of rs5743795 might play a protective role in SAP (CC OR = 2.426, *P* = 0.015 and GG OR = 0.909, *P* < 0.05, resp.). The polymorphisms of the TLRs contribute to the development of severe AP.

#### 4.1.2. CD14

CD14 is a type of pattern recognition receptor that is primarily expressed on the cell surface of monocytes and phagocytes [61]. As a coreceptor of TLRs, CD14 binds lipopolysaccharide (LPS) to the LPS receptor complex, eventually activating a series of inflammatory reactions [62]. The soluble subtype of CD14, sCD14, was shown to increase the mediation of the systemic inflammatory response to AP [63]. A recent paper indicated that the *CD14*-651C/T (dbSNP: rs5744455) polymorphism, a promoter polymorphism, was correlated with the severity of AP in Japanese patients (SAP versus control *P* = 0.005 and SAP versus MAP *P* = 0.001) [64]. A plausible mechanism for the effect of this mutation on AP is the promotion of gene transcription and an increase in the serum level of sCD14, both of which may contribute to the severe inflammatory cascade during AP.

#### 4.1.3. Monocyte Chemoattractant Protein-1 (MCP-1)

Chemokines are a subfamily of cytokines that play critical roles in inducing leucocytes to migrate to the inflammatory site [65]. Experimental models have shown that MCP-1, a subtype of the C-C chemokine, can be an important serum marker in evaluating the severity of AP [66]. Investigators from America and China have shown that the *MCP-1*-2518 (rs1024611) G allele can aggravate the severity of or the susceptibility to AP by elevating the serum level of MCP-1 [67, 68]. Further investigations are needed to confirm the role of this mutation in AP because of the limited size of the two studies.

#### 4.1.4. Human Beta Defensins (HBDs)

HBDs, which are derived from intestinal mucosal epithelial cells, can directly kill invading pathogens and maintain the integrity of the intestinal barrier. HBD-1 and HBD-2, encoded by the *DEFB1* and *DEFB4* genes, respectively, are considered to be associated with AP [69, 70]. A study from Hungary revealed that *DEFB1* and *DEFB4* are relevant to SAP [69]. The investigators discovered three SNPs in the *DEFB1* gene [G–20A (c.–20G→A) (rs11362), G–52A (c.–52G→A) (rs1799946), and C–44G (c.–44C→G) (rs1800972)] that may be associated with SAP (*P* = 0.009, 0.03, 0.001, resp.), with the first two SNPs showing that the AA genotype is a risk factor and the third SNP showing that the GG genotype is a protective factor. In addition, they found that these pathogenic variants may enhance the risk of bacterial infection during AP, a finding that is consistent with the main function of the proteins. A meta-analysis of the *DEFB1* gene effect on digestive diseases confirmed that these three *DEFB1* SNPs are strongly correlated with digestive diseases, including SAP [70].

#### 4.1.5. Mannose-Binding Lectin (MBL)

MBL belongs to the subgroup of collectins in the C-type lectin superfamily that acts as the first defense by taking up pattern recognition proteins participating in the innate immune response [71]. By recognizing and binding carbohydrate structures from microbial surfaces, MBL can activate complement in an antibody- and C1q-independent manner [72], followed by a series of inflammatory responses. Previous studies have identified *MBL2* alleles that are correlated with some infectious diseases and accompanied by decreased serum levels of the protein [73, 74]. Zhang et al. [75] were the first to discover that the *MBL2* HY/LX genotype (one kind of haplotype) was associated with the severity of AP (SAP 26% versus MAP 14%, *P* = 0.028). This result requires further research for confirmation.

### 4.2. Susceptibility Genes Contributing to the Complications of the Disease

#### 4.2.1. TNF-*α*

**Figure 2:**
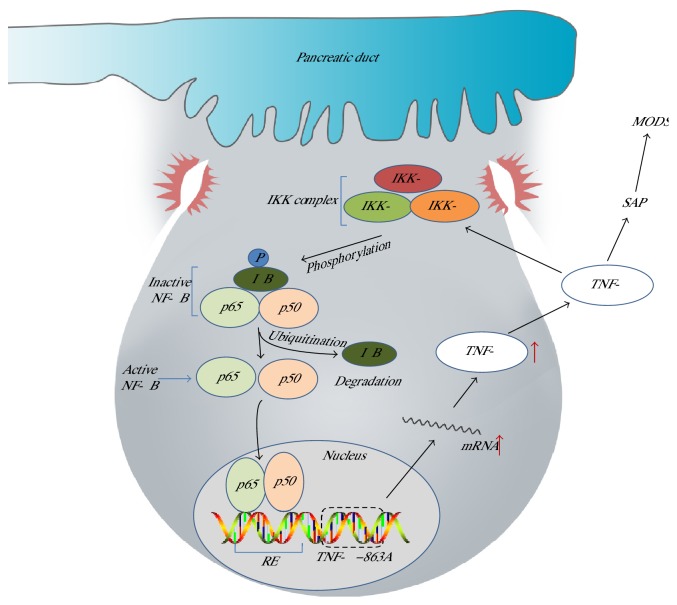
A hypothesis of the interaction between NF-*κ*B pathway activation and *TNF-α* −863A allele polymorphism.

TNF-*α* acts as a key proinflammatory mediator, activating the NF-*κ*B signaling pathway and subsequently elevating the level of other cytokines and chemokines under stress. This action forms a positive feedback loop that aggravates the disease. Several studies found that TNF-alpha gene polymorphisms were correlated with AP-related complications, including MODS, SIRS, and septic shock [76–80]. Bishehsari et al. [76] discovered that the *TNF-α* −1031C (rs1799964) and −863A (rs1800630) alleles significantly increased the risk of AP progression to MODS (56.5% versus 32.4%, *P* = 0.022 and 43.5% versus 21.8%, *P* = 0.022, resp.) (Figure 2). A study of the Han Chinese population found that the rs5029924 polymorphism of TNF-*α*-induced protein 3 (*TNFAIP3*) could increase the susceptibility of AP patients to SIRS by elevating the serum level of A20 protein [80]. Zhang et al. [77–79] published several papers suggesting that the *TNF2* allele (*TNF-α* −308A) is associated with death as a result of SAP-associated septic shock, but the polymorphism may not function because no significant differences were found in the serum levels of TNF-*α.* In spite of the critical role of TNF-*α* in AP, it can hardly be a marker because of the short half-life. However, targeted therapy can be adapted to inhibit different systematic complications to which TNF-*α* predisposes.

Environmental factors (e.g., alcohol and gallstone) stimulate proinflammatory cytokines in vivo (including TNF-*α* and IL-1*β*), activating the classical NF-*κ*B signal pathway. Subsequently, the DNA/NF-*κ*B complex impels *TNF-α* −863A expression and then elevates the levels of mRNA and TNF-*α*, forming a positive feedback loop. Increasing TNF-*α* exacerbates the disease to SAP and eventually leads to MODS and death. IKK refers to I*κ*B kinase; RE refers to response elements.

#### 4.2.2. IL-10

As described above, IL-10 acts as an anti-inflammatory cytokine that lowers cell-mediated inflammatory responses, and it is implicated in immunosuppression in CARS. Several studies showed no significant correlation of the IL-10 gene polymorphism with AP or SAP [36, 81, 82]. Interestingly, one of the studies showed that *IL-10*-1082G may increase the susceptibility of SAP patients to septic shock (*χ*^2^ = 5.921, *P* = 0.015) [81].

#### 4.2.3. TLR4

TLR4 is a vital transmembrane receptor that processes LPS and is absolutely necessary for the subsequent signal pathway that results in an effective host defense. Gao et al. [83] indicated that the *TLR-4* Asp299Gly polymorphism increased the tendency of AP patients to develop pancreatic necrotic infection caused by gram-negative bacteria (*P* = 0.03).

## 5. Conclusion

Environmental factors (e.g., ethanol, duct bile, and metabolites) injure acinar cells and cause AP. Genetic variants may strengthen or amplify the role of environmental factors and make individuals predisposed to the disease. Based on the mechanisms involved and the effects on the progression of the disease, we classified the genes into early- and late-stage related genes. The genes can also be divided into pancreatic tissue-specific and nonspecific genes. For the early-stage group, the susceptibility genes are mainly associated with the occurrence of AP with or without different etiology. For the late-stage group, the susceptibility genes are mainly associated with the severity of AP or with severe complications. What is noteworthy is that the three pancreas-specific gene variants are all associated with the early stage of pancreatitis. This association is consistent with the experimental data suggesting that it is the inflammatory pathway signaling rather than premature trypsinogen activation that results in the severe inflammatory response in AP. Furthermore, there are susceptibility genes of the inflammatory signaling pathway involved in the early stage of pancreatitis, supporting the important role of NF-*κ*B activation in AP.

Most cases of AP present a reversible process, suggesting that the genetic factors may act as modifiers that predispose individuals exposed to environmental factors to AP. The interactions deserve attention because the effects of specific genetic variants may vary with different environmental factors (e.g., alcohol), and an understanding of the interactions will help clinicians remove inducing factors. Focusing on gene polymorphisms on AP may also contribute to change final treatment decisions. For instance, the role of susceptibility genes in late-stage AP is helpful as it explains methodically what clinicians see commonly and, when logically extended, argues against empiric use of antibiotics early in acute pancreatitis as a response to SIRS. In addition, interactions of multiple genes, for example, the coexistence of *CTRC* and *SPINK1* mutations, may aggravate the disease. Since severe cases of AP are life-threatening, a better understanding of how genetic factors affect the progression of AP and lead to a severe disease or to severe complications will be conducive to timely interventions and targeted therapies. Although most of the susceptibility genes are disease-nonspecific, they potentially contribute to a poor prognosis. Molecular targeted therapy is no longer a fiction for AP patients, especially for severe cases.

Overall, in this review, we have combined the current understanding of AP pathogenesis with variants of susceptibility genes to provide a novel perspective on the disease. As an emerging field, the effects of genetic polymorphisms on AP are worth further study and may provide a distinctive perspective on the disease.
